# Assessing Deceleration Performance: Methodological and Practical Considerations

**DOI:** 10.1007/s40279-025-02339-7

**Published:** 2025-11-27

**Authors:** Damian J. Harper, Nicolas M. Philipp, Ola Eriksrud, Paul A. Jones, Philip Graham-Smith, Thomas Dos’Santos

**Affiliations:** 1 Institute of Coaching and Performance (ICaP), School of Health, Social Work and Sport, University of Lancashire, Preston, PR1 2HE UK; 2 Denver Nuggets Basketball Club, Denver, CO USA; 3 Biomechanics Laboratory, Department of Physical Performance, Norwegian School of Sport Sciences, Oslo, Norway; 4 Directorate of Psychology and Sport, University of Salford, Salford, Greater Manchester UK; 5 Department of Sport and Exercise Sciences, Manchester Institute of Sport, Manchester Metropolitan University, Manchester, UK

## Abstract

Deceleration is a critical locomotor skill for athletes competing in multi-directional speed sports. Greater deceleration can help athletes perform rapid reductions in velocity facilitating rapid changes of direction, whilst the high mechanical forces associated with braking can be linked to a heightened risk of fatigue, tissue damage and injuries. Despite the clear importance of deceleration in sport, research and applied practices in the past have predominantly focused on assessing an athlete’s sprint acceleration and maximum velocity capabilities, neglecting the necessity to be able to decelerate. With tactical evolutions in sports demanding athletes to accelerate and attain higher sprinting speeds more frequently in competition, there is increased necessity to decelerate and to be able to accurately assess this movement skill. Therefore, the aim of this article is to discuss methodological and practical considerations of the protocols and measurement technologies that can be used to assess deceleration in an applied field-based environment. The article highlights a range of different protocols (i.e. change of direction and acceleration-deceleration ability tests) and measurement technologies (i.e. radar, laser, video, global navigation satellite systems, inertial measurement units and motorised resistance devices) that can be used to evaluate deceleration and some of the advantages and disadvantages of each. Key metrics used to measure deceleration performance, and the kinematics underpinning deceleration technique are highlighted. Given the performance, health and injury-risk implications associated with deceleration, assessment of this movement skill should be given high priority within any athlete multi-disciplinary support system.

## Key Points

**Table:** 

The focus in sports performance has been on assessing and developing acceleration and maximum velocity sprinting capabilities, neglecting the necessity to be able to decelerate
The challenges for assessment of deceleration qualities are to provide some control by standardising acceleration distance, or total distance and expressing deceleration performance (in terms of deceleration distance [m], deceleration time [s] or deceleration [m/s^2^]) in the context of the maximum velocity attained in the acceleration phase
Deceleration tests must be designed so that the velocity of the athlete drops to an instantaneous zero, which provides a definitive endpoint to the deceleration task. This can include ‘acceleration to stop at a point’, or ‘acceleration-deceleration to re-acceleration in 90° to 180° turns. All of these must be measured using a validated device that can measure instantaneous velocity
The commands must ensure the necessity to accelerate to the highest possible velocity within the constraints of the test (i.e. set distance). Inherently, this velocity and momentum will be different for all athletes
Designing deceleration test batteries that require each leg to act as both penultimate and final contact limbs can give further insights into preferential load distribution and identify deficiencies in lower extremity strength and co-ordination

## Introduction

In multi-directional speed (MDS) sports (e.g. soccer, American Football, basketball, rugby), athletes must frequently change velocity to manoeuvre safely and effectively within the constraints of their competitive environment. These changes in velocity require athletes to accelerate (i.e. increase velocity) and decelerate (i.e. decrease velocity) across varying distances and times. For this article, both ‘acceleration’ and ‘deceleration’ refer to any human locomotor action that requires an increase or decrease in running velocity, respectively, and that are self-initiated without the use of force being imparted by other individuals (e.g. being hit or tackled). Attaining superior acceleration and deceleration capabilities is of paramount importance to athletes competing in MDS sports as it can provide them with the physical resources to outmanoeuvre their opponents, leading to successful match performance outcomes [1]. Therefore, accurately profiling these capabilities is also of significant interest to sports science, strength and conditioning, performance and healthcare professionals working with MDS sport athletes.

For assessment of sprint acceleration, there are evidence-based guidelines available for practitioners on the methodological considerations (e.g. choice of technology, start positions) required to establish and implement standardised, reliable and valid testing protocols [2, 3]. To the authors’ knowledge, however, none currently exist for assessing deceleration. A major reason for this, historically, has been the difficulty in assessing deceleration in an applied field-based environment in comparison to acceleration, where surrogates of performance can simply be attained using split-times collected via electronic timing gates across different distances [2, 4]. This is not the case for deceleration, where electronic timing gates may only permit assessment of indirect indices [5, 6] or broad estimates of deceleration ability if multiple electronic timing gates are spaced closely together [7]. Therefore, research and applied practices have been dominated by evaluating the more easily measurable components of performance that rely predominantly on the generation of propulsive forces (i.e. acceleration). Subsequently, knowledge about the determinants of athletic performance and the most effective training methods have been biased towards getting athletes faster (i.e. greater acceleration and maximal velocity sprinting capabilities) [8]. We argue that the traditional pursuit of increasing athletes’ speed in particular contexts could in fact be harmful if the athlete does not have the physical capacity to decelerate from higher entry momentums and tolerate the associative forces. Therefore, there is a self-regulatory motor control aspect where athletes will control their velocity based on their perceived ability to decelerate and transition into the next movement.

For MDS sports, obtaining accurate information on an athlete’s deceleration capabilities combined with their acceleration capabilities is critical [8, 9]. High-intensity decelerations are performed frequently in many MDS sports [10], are the locomotor action that can generate the greatest changes in velocity [11] and can subsequently expose athletes to some of the highest mechanical forces they will encounter during competition [12–18]. Therefore, decelerating can expose athletes to a heightened risk of lower extremity injuries (e.g. anterior cruciate ligament [ACL] rupture) [19–22], and be particularly sensitive to neuromuscular fatigue and mechanically induced tissue damage [23–25] because of substantial negative work demands that require eccentric muscle contractions (i.e. active muscle fascicle lengthening) to absorb kinetic energy [16, 26]. With tactical evolutions in MDS sports requiring athletes to accelerate and attain higher sprinting speeds more frequently in competition, there is consequently increased importance and demand for decelerating more frequently, and at higher intensities, effectively [27]. Additionally, accurately profiling player deceleration capabilities can also help monitor and inform performance [8], rehabilitation [28] and injury-risk reduction [9] training programmes.

Developments in field-based technologies have enabled deceleration assessments to be more accessible to practitioners and for researchers to investigate the reliability [29–31] and validity [32, 33] of different measurement devices, testing protocols and performance metrics. However, there are currently no evidence-based guidelines that summarise methodological considerations to establish a standardised, reliable and valid assessment of an athlete’s deceleration capabilities. Given the performance, health and injury-risk implications associated with deceleration, assessment of this movement skill should be given high priority within any athlete multi-disciplinary support system. Therefore, the aims of this article are to: (a) provide an overview of different protocols that can be used to assess deceleration; (b) discuss different technologies that can be used to assess deceleration and the advantages and disadvantages of each; (c) discuss common metrics used to assess deceleration and provide normative values across different sports; and (d) discuss potential implications for applied practices gleamed from assessing an athlete’s deceleration capabilities.

## Protocols for Assessing Deceleration

**Fig. 1:**
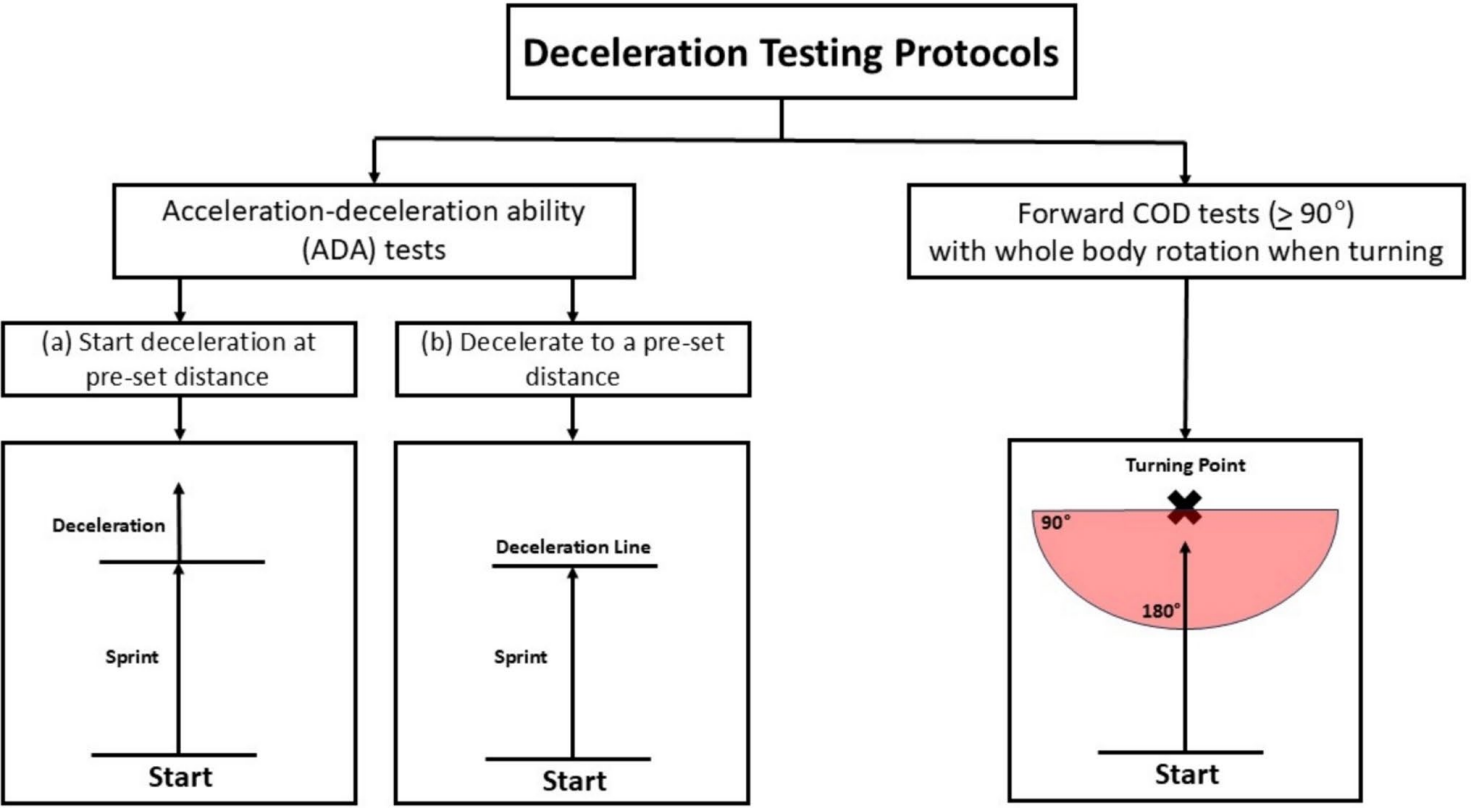
Testing protocols used to assess deceleration. *COD* change of direction. Note: *Red area* indicates the exit area following the turning point for forward COD tests

Deceleration in MDS sports is performed as an isolated agility action, or when preceding a directional change [34]. Therefore, to assess deceleration, two types of testing protocols can be selected (Fig. 1). These include acceleration-deceleration ability (ADA) tests [29, 35] or change of direction (COD) tests [31, 33, 36, 37] with angles greater than 90° requiring whole body rotation. Importantly, both test options require whole body velocity to be momentarily reduced to zero in the initial direction of travel. Because of the importance of accurately assessing deceleration, the subsequent sections of this article will only incorporate recommendations from studies that have directly measured deceleration kinetics or kinematics during ADA or COD tests encompassing angles > 90°. More acute COD angles (i.e. < 90°) are not of interest because these angles have lower deceleration demands and there is more reliance on maintaining velocity throughout the turn [38].

### Acceleration-Deceleration Ability Tests

For the ADA tests, two test protocols are available: (a) start deceleration at a pre-set distance or (b) decelerate to a pre-set distance (Fig. 1). With each option, the entry velocity at which deceleration (i.e. braking) commences can be modified based on the sprint approach distance selected, with longer approach distances requiring greater deceleration distances, times to stop and number of braking steps [35, 39]. For ADA tests, athletes are instructed to come to a static stop or to perform a subsequent action post deceleration that avoids whole body rotation movements required in sharp COD tests (Sect. 2.2). For example, in sports such as basketball and American Football, having athletes backpedal a specific distance or number of steps post deceleration may better mimic movement actions common in their game. Furthermore, to instigate an unanticipated deceleration, practitioners may also consider instigating deceleration with an unplanned stimulus (e.g. sound, light or human), although currently to the authors’ knowledge, no research has investigated the reliability of such a protocol. It is also important to note that prior to any ADA test, a familiarisation session is recommended to help reduce any potential learning effects that could be associated with the test [29].

In the original ADA test protocol devised by Harper et al. [29], athletes were required to sprint 20 m prior to decelerating before backpedaling back to the 20-m line (Fig. 1a). Furthermore, to help reduce any potential pacing strategy prior to decelerating and to standardise the distance at which deceleration commences, athletes had to attain within 5% of their 20-m sprint time without a maximal deceleration. In the authors’ experience, if using this approach with large groups of athletes, an automated detection of the required threshold would be recommended. The original 20-m ADA test sprint distance has also been used in other studies [40–44] with modifications of this protocol including 4.5-m [45], 5-m [43, 46], 9.14-m (10 yards) [39, 47], 10-m [43, 46–52], 15-m [5, 30, 53, 54], 18.29-m (20 yards) [39] and 30-m [55, 56] sprint approach distances. Whilst deceleration is generally self-initiated at the marked pre-set sprint distance (i.e. braking line), timing cells have also been used to administer an audible cue to signify crossing of the deceleration line, thus further helping to ensure deceleration commences at the required distance [30, 50, 52]. Nonetheless, it is possible deceleration could commence prior to the marked deceleration line [29], emphasising the importance of deceleration being measured using instantaneous velocity and from the timepoint immediately preceding the maximum approach velocity prior to decelerating (Sect. 4.1).

Acceleration-deceleration ability tests requiring athletes to stop at a pre-set distance (Fig. 1b) have been used by several studies to assess deceleration performance outcomes [35, 45, 57–59] and surrogates of injury risk [60]. Graham-Smith et al. [35] examined sprint-to-stop distances of 5 m, 10 m, 15 m and 20 m, with each incremental sprint distance requiring greater deceleration distances (5 m = 2.93 ± 0.12 m, 10 m = 4.94 ± 0.39 m, 15 m = 6.61 ± 0.40 m, 20 m = 7.93 ± 0.62 m). Other sprint-to-stop distances have included 4.5 m [59], 4.6 m [60] and 13 m [57, 58], although it should be noted that all these studies focused on the final braking step, with one study calculating deceleration (m/s^2^) across a 1-m distance preceding the final foot braking step [59].

### Change of Direction Tests

**Fig. 2:**
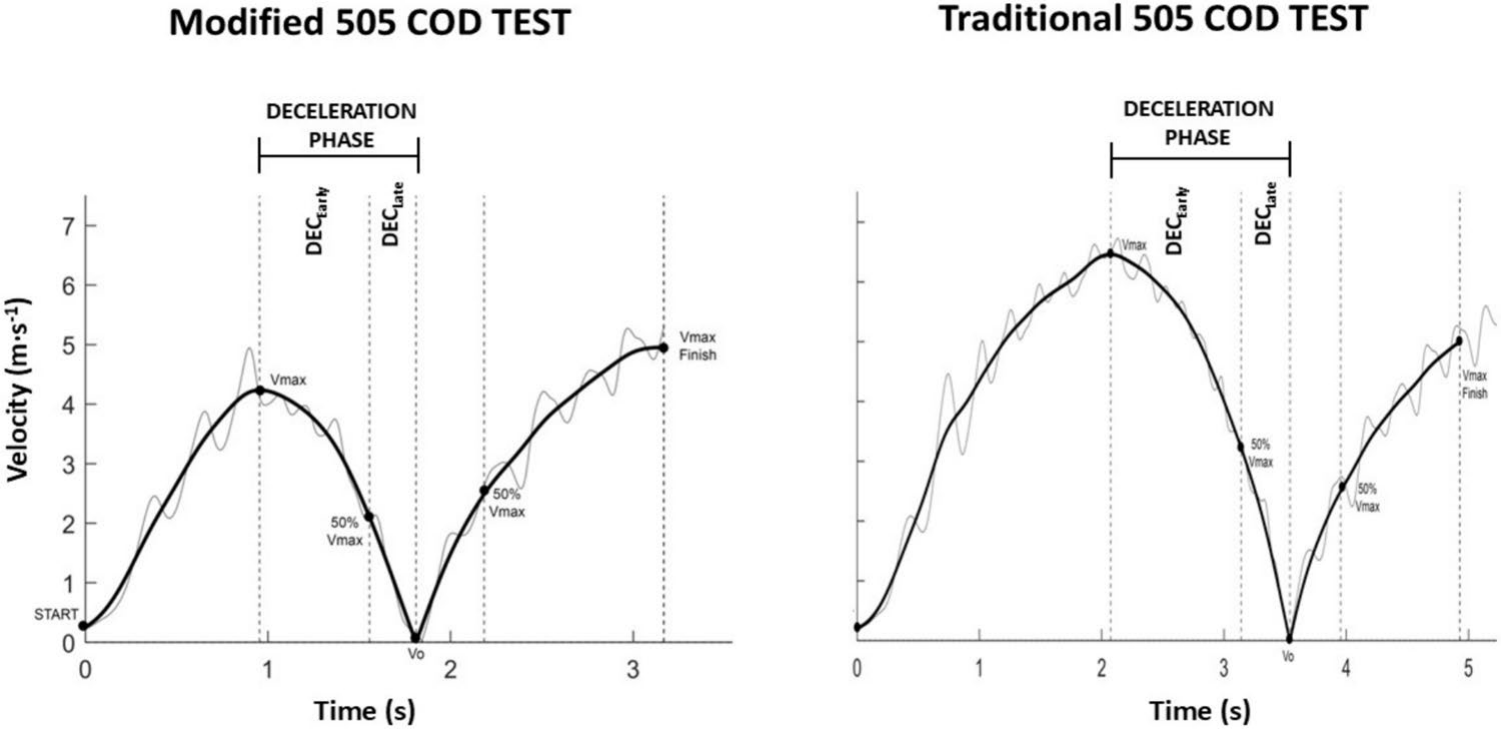
Instantaneous velocity–time profiles in modified 505 (lower entry velocity) and traditional 505 (higher entry velocity) change of direction (COD) tests, enabling the identification and evaluation of deceleration phase-specific performance. *DEC*_*Early*_ early deceleration sub-phase, *DEC*_*Late*_ late deceleration sub-phase, *Vmax* maximum velocity, *V*_*0*_ zero velocity

In sharp COD tests (i.e. 90°–180°) requiring whole body rotation, substantial braking over multiple foot contacts is required during the deceleration phase to momentarily reduce velocity to zero prior to turning [38]. Therefore, sharp COD tests are another option for assessing deceleration capabilities. Traditionally, however, completion time is a commonly used metric for a COD assessment, which is an oversimplification of COD ability, and fails to provide information specifically related to the deceleration phase. Thus, technologies (Sect. 3) that permit instantaneous measures of the horizontal velocity of the centre of mass (COM) over key phases of the COD (i.e. acceleration, deceleration and re-acceleration phase-specific information) can provide a more holistic overview of deceleration and COD ability, helping to identify strengths and areas for development (Fig. 2). Sharp COD tests including the modified (i.e. 5-m approach prior to 180° turn) and traditional 505 (i.e. 15-m approach prior to 180° turn) are tests that simultaneously evaluate deceleration and COD abilities. These COD tests also make it possible for practitioners to evaluate deceleration from different approach distances and speeds that may best reflect the positional COD demands of the sport [61]. Currently, however, only a limited number of studies have provided phase-specific or instantaneous velocity information during 505 COD tests using radar [48], laser [37, 62], inertial measurement units [50, 52] and motorised resistance [31, 33, 63–65] technologies. Nevertheless, these studies highlight technological solutions that can be used to gain advanced insights into deceleration abilities (e.g. average and peak deceleration, deceleration distance and time, and braking ground contact time) during COD assessments, although further research is needed providing normative 505 COD phase-specific data across various populations.

Generally, there is limited research pertaining to phase-specific and instantaneous velocity measures during multiplanar tasks such as CODs of 90°–135°, which are not uniplanar [36, 66] but may help to enhance ecological validity in some sports. This is likely owing to some technologies being restricted to tracking athletes in one plane (i.e. directly behind or in-front of COM), such as with some radar and laser devices. Advancements in technologies (Sect. 3) offer solutions for evaluating deceleration abilities across a spectrum of COD angles by tracking COM trajectory data (i.e. *x*, *y* coordinates). The reliability, validity and application of these devices, however, still needs to be established in sporting practice. Similar to ADA tests, to help enhance reliability and reduce potential learning effects associated with any sharp COD test, it is recommended to include a familiarisation session prior to testing. A summary of considerations when choosing a deceleration test is illustrated in Table 1.

**Table 1:** Summary of considerations when choosing a deceleration test

Considerations	Importance
Choice of test (i.e. ADA or COD test)	ADA tests allow athletes to decelerate without the necessity to perform a turn with whole body rotation. This potentially reduces task complexity, a factor that has also been highlighted for assessing deceleration when walking [67] During COD tests, pure deceleration qualities may be contaminated by other factors such as the skill required to execute a turn with whole body rotation prior to re-acceleration COD tasks generally involve deceleration in a rotated position over multiple foot contacts to reduce the redirection demands and may permit a different evaluation of deceleration ability, which involves a different braking strategy [50] Consequently, based on the above point, use of both an ADA and COD test may allow simultaneous evaluation of deceleration and COD ability ADA tests may be a more suitable option particularly during earlier phases of field-based rehabilitation to evaluate an athlete’s ability to safely decelerate prior to COD [68, 69]. Subsequently, COD tests could be integrated in the periods prior to and following return to sport Using ADA and COD tests that require each leg to act as both penultimate and final contact limbs can give further insights into preferential load distribution and identify deficiencies in lower extremity strength and motor coordination
Sprint approach distance/speed	Shorter sprint distances will require deceleration to be initiated from lower percentages of maximal sprinting speed/momentum and therefore require deceleration to be performed across fewer steps, and less distance and time Deceleration kinetic and kinematic demands can be substantially different when performing decelerations from lower (e.g. 10-m approach) compared to higher (e.g. 20-m approach) sprinting speeds [39], meaning athletes could have good deceleration performance from lower sprinting speeds, but not higher sprinting speeds, and vice-versa [39]. This may therefore warrant assessment of deceleration from both lower and higher sprinting velocities When decelerating from higher sprinting velocity/momentum, the early braking steps require forces to be generated with single-limb support across very short time frames, and can have high-impact peak forces and loading rates [8]. Thus, during early phases of field-based rehabilitation, selecting shorter sprint approach distances may help to reduce these demands
Position and sport-specific sprint distances	Selecting a deceleration test and sprint approach distance that best reflects the positional demands of the sport [61] In sports and positions where decelerations are performed from a wide range of sprint velocities, a deceleration assessment may need to be conducted using a test that requires deceleration from both lower and higher sprint velocities

*ADA* acceleration-deceleration ability, *COD* change of direction

## Technological Considerations when Assessing Deceleration

The following section provides an overview of considerations when assessing deceleration with different field-based technologies including radar/laser, motorised resistance devices (MRDs), global navigation satellite system (GNSS) and local positioning systems, video and inertial measurement units (IMUs). To ensure consistent interpretation of reliability data amongst studies and technology devices, relative reliability (i.e. intra-class correlation [ICC]) is interpreted using the guidelines from Koo and Li [70] as: poor (≤ 0.49), moderate (0.50–0.74), good (0.75–0.89) and excellent (≥ 0.90) with absolute reliability (i.e. co-efficient of variation; [CV%]) interpreted using recommendations of McMahon et al. [71] as: poor (> 15%), moderate (10–15%), good (5–10%) and excellent (< 5%).

### Radar/Laser Devices

The ability to measure instantaneous velocity and distance makes the use of laser and radar devices appealing for acceleration, maximum speed and deceleration assessment [3]. Lasers have been used in several studies to measure deceleration abilities using the LAVEG (LAser VElocity Guard; Jenoptik Technologies, Jena, Germany) [30, 35–37, 66] or MuscleLab LaserSpeed (Erotest Innovation AS, Stathelle, Norway) [62, 72] laser devices, both sampling at 100 Hz. However, to the author’s knowledge, only two studies [30, 36] have investigated the reliability of deceleration performance metrics established from the LAVEG laser device. Ashton and Jones [30] investigated the intra-session and inter-session reliability of the distance required to decelerate from 75%, 50%, 25% and 0% of the 15-m sprinting speed reporting moderate-to-good (ICC = 0.64–0.83) and good-to-excellent (ICC = 0.79–0.97) intra-session and inter-session relative reliability, respectively. The CV%, however, for these metrics ranged between moderate and poor (11–17%), making it difficult to detect small changes in deceleration performance. Similarly, Hader et al. [36] reported a CV% of 38% and 13% for peak deceleration (m/s^2^) and distance at peak deceleration (m), respectively, when captured during a 90° COD test calculated from metre-to-metre changes in speed over time. Currently, no study has evaluated the validity or reliability of a laser device for measuring instantaneous deceleration or evaluated average deceleration metrics (m/s^2^) captured from across the whole of the deceleration phase.

The only radar device that has been used by numerous studies to evaluate deceleration performance metrics is the Stalker ATS II (Stalker Sport, Richardson, TX, USA), which samples at 47 Hz [29, 43, 44, 46–48, 50, 53–55, 73]. The reliability of this device for measuring deceleration has been reported in numerous studies [5, 29, 43, 44, 47] with intra-session reliability reported between moderate and excellent (CV% = 1.5–16.7%) for a range of whole body deceleration performance metrics (i.e. average and maximum deceleration, early and late deceleration, distance and time to stop). Practitioners should be aware, however, that data captured with the Stalker ATS II require manual data processing procedures and that reliability could be affected if different individuals process the data [47]. Therefore, if using this device, it is recommended to use one trained individual and ideally have a fully automated processing procedure using selected filters and algorithms that would negate the need to establish inter-rater reliability [47]. Currently, only one study to the authors’ knowledge has used radar to assess deceleration during a COD test (traditional 505), but the CV% for deceleration metrics ranged between 11 and 15% [48]. Advancements in radar-based three-dimensional sensor technology (e.g. Photon Sports; Ledsreact, Kortrijk, Belgium), however, permit future opportunities to investigate the validity, reliability and application of deceleration assessment in both uni- and multi-planar COD tasks.

### Motorised Resistance Devices

Motorised resistance devices use engines to provide resistance rather than weights, bands or pressure. Specifically, the computer-controlled engine allows for precise prescription of resistance, speed and how the resistance behaves (i.e. isoinertial, isotonic) in different movement directions (i.e. concentric/resisted and eccentric/assisted). Both the control (i.e. load, speed) and presentation of continuous data (i.e. velocity, force, power) are achieved at high frequencies (> 200 Hz). This ensures rapid changes of athlete movement velocity during deceleration and COD can be detected, ensuring accuracy of measurements. There are different types of machines available that can be grouped into gym-based and field-based machines. In general, field-based machines have longer lines, greater speeds and lower resistances in comparison with gym-based machines. If tests are to be done beyond one or two deceleration steps, field-based machines should be employed as both line length and speed capacities (14 m/s) will not be a limiting factor when performing tests such as ADA or COD.

For the purposes of assessing deceleration, an MRD (1080 Sprint, 1080 Motion, Sweden) sampling at 333 Hz has been reported to be valid for measuring velocity before (− 1.5 s) and after (+ 1.5 s) a 180° turn during a modified 505 COD test when compared to a three-dimensional (3D) motion analysis system [33]. Furthermore, the reliability of the same MRDs for measuring various deceleration metrics has been investigated in 180° COD tests of varying distances (i.e. 5-m, 10-m and 15-m approaches) with left and right foot turns [31] and in a 30-m ADA test [56]. In the study by Westheim et al. [31], almost all deceleration metrics had good-to-excellent absolute reliability across each COD test. Furthermore, West et al. [56] reported good absolute (CV% = 5.3–7.1%) and excellent relative (ICC = 0.92–0.97) inter-session reliability when assessing a range of key deceleration metrics (i.e. average deceleration, distance and time to stop) using a threshold based approach (i.e. deceleration starts from first value ≤ 1.5 m/s^2^) averaged across the best of two trials in a 30-m ADA test. Collectively, these findings highlight the potential of the 1080 Sprint for assessing deceleration performance during COD and ADA tests. Future research is needed, however, to investigate the validity and reliability of the 1080 Sprint for assessing deceleration performance in ADA tests with shorter sprint distances (i.e. 5, 10, 15 and 20 m), and to evaluate the validity and reliability of other MRDs (e.g. DynaSpeed, Ergotest Innovation AS, Langesund, Norway) for assessing deceleration performance during sharp COD and ADA tests.

### Global Navigation Satellite and Local Positioning Systems

Numerous studies have evaluated the validity and reliability of GNSS [74–82] and local positioning systems [83, 84] for assessing sprint acceleration and maximum velocity sprinting capabilities, confirming their potential for assessing these performance capabilities. Conversely, to the authors’ knowledge, there is currently only one study that has examined the validity and reliability of a GNSS device (10 Hz Apex; STATSports, Newry, UK) for the purpose of assessing deceleration during an ADA test with a 20-m sprint approach distance [44]. Based on agreement with radar evaluated with equivalence testing (i.e. values within the smallest effect size of interest), the findings of this study support the use of average deceleration and maximal deceleration metrics, with deceleration distance and time to stop metrics outside of equivalence bounds. It is important to note that only raw GNSS data permit calculation of average deceleration, which requires additional processing procedures and filtering approaches, such as a digital fourth-order Butterworth filter as used by Jones et al. [44]. Indeed, compared with other filtering approaches, use of a fourth-order Butterworth filter with a cut-off frequency of 2 Hz has also been reported to have the best level of agreement, accuracy and precision between a GNSS device (Vector S7; Catapult Sports) and a 100-Hz Vicon 3D motion analysis system for the purpose of calculating deceleration during sprint-to-stop motions and 90° and 180° COD manoeuvres [85]. Therefore, based on the findings of these studies, and for the purpose of assessing deceleration with GNSS devices, it is recommended for practitioners to use their own custom processing of the exported raw data using alternative filtering approaches, and to carefully examine the reliability and validity of selected metrics.

### Video

Although 3D motion capture is considered the gold standard for evaluating COM velocity and joint kinematics, the technology is expensive and generally restricted to laboratory environments. Thus, two-dimensional (2D) high-speed camera-based technology, which is a cheaper and accessible feature of most smartphones and tablets, can be used to derive instantaneous measures of COM velocity in the field, or to measure key deceleration indicators such as distance and time to stop [86] with a camera positioned perpendicular to the deceleration plane of motion. Advancements in computer vision technology (i.e. marker-less tracking) and artificial intelligence provide opportunity to process 2D video data quickly to attain frame-by-frame kinematic information on deceleration performance, overcoming time-consuming processes and feedback delays often associated with manual 2D video digitisation [87]. However, the validity and reliability of data captured using this technology require further research [88]. In addition to whole body deceleration-related metrics (i.e. average, peak deceleration), video provides important visual records of kinematic data (e.g. step lengths, joint angles/angular velocities) helping to evaluate the athlete’s deceleration technique (Sect. 4.2), which could have important implications for performance enhancement (Sect. 5.1) and rehabilitation and injury-risk reduction procedures (Sect. 5.2). Because deceleration has the propensity to generate very large forces, a sub-optimal technique could further amplify tissue damage and injury risk as highlighted in previous studies investigating joint kinematics associated with peak ground reaction forces [60], increased knee joint loading [89] and future ACL injury risk [51] during maximal deceleration to stop tests (i.e. ADA tests). Therefore, practitioners are recommended to record 2D sagittal plane video footage and to select deceleration technical metrics (Sect. 4) that best represent superior deceleration performance and movement quality.

### Inertial Measurement Units

Inertial measurement units are devices that contain sensors that can perceive movement in multiple dimensions tracked on a cartesian coordinate system (i.e. *x*-axis, *y*-axis and *z*-axis). As this relates to assessing whole body deceleration and kinetic and kinematic variables during ADA and sharp COD tests, to the authors’ knowledge, only a few studies have done so using IMUs [5, 32, 50, 90–92]. Large to almost perfect associations (*r* = 0.65–0.98) and small-to-trivial effect size differences (0.57–0.09) have been reported between an IMU system (Xsens MVN; Xsens, Enschede, the Netherlands) consisting of units attached to the feet, shanks, thighs, and pelvis and a 3D motion capture system for measuring deceleration hip and knee joint kinematics (i.e. joint angles and angular velocities) and spatial–temporal (i.e. ground contact time and touchdown distance) variables at lower intensities (i.e. 50% effort) [32]. With increases in approach velocity (i.e. 100% effort), however, only ground contact time and knee flexion angular velocity had both very large associations (*r* ≥ 0.84) and trivial-to-small effect size differences (0.07–0.27) between systems [32]. Using the same IMU system, Philipp et al. [50] compared the horizontal deceleration demands between an ADA test with a 10-m sprint approach and a traditional 505 COD test. The authors reported significant differences in deceleration demands (i.e. average deceleration, deceleration time and distance, ground contact times and touchdown distance) between the two assessments, with intra-session relative reliability that ranged from poor to excellent (ICC = 0.13–0.98) based on the test and or metric of interest [50].

**Fig. 3:**
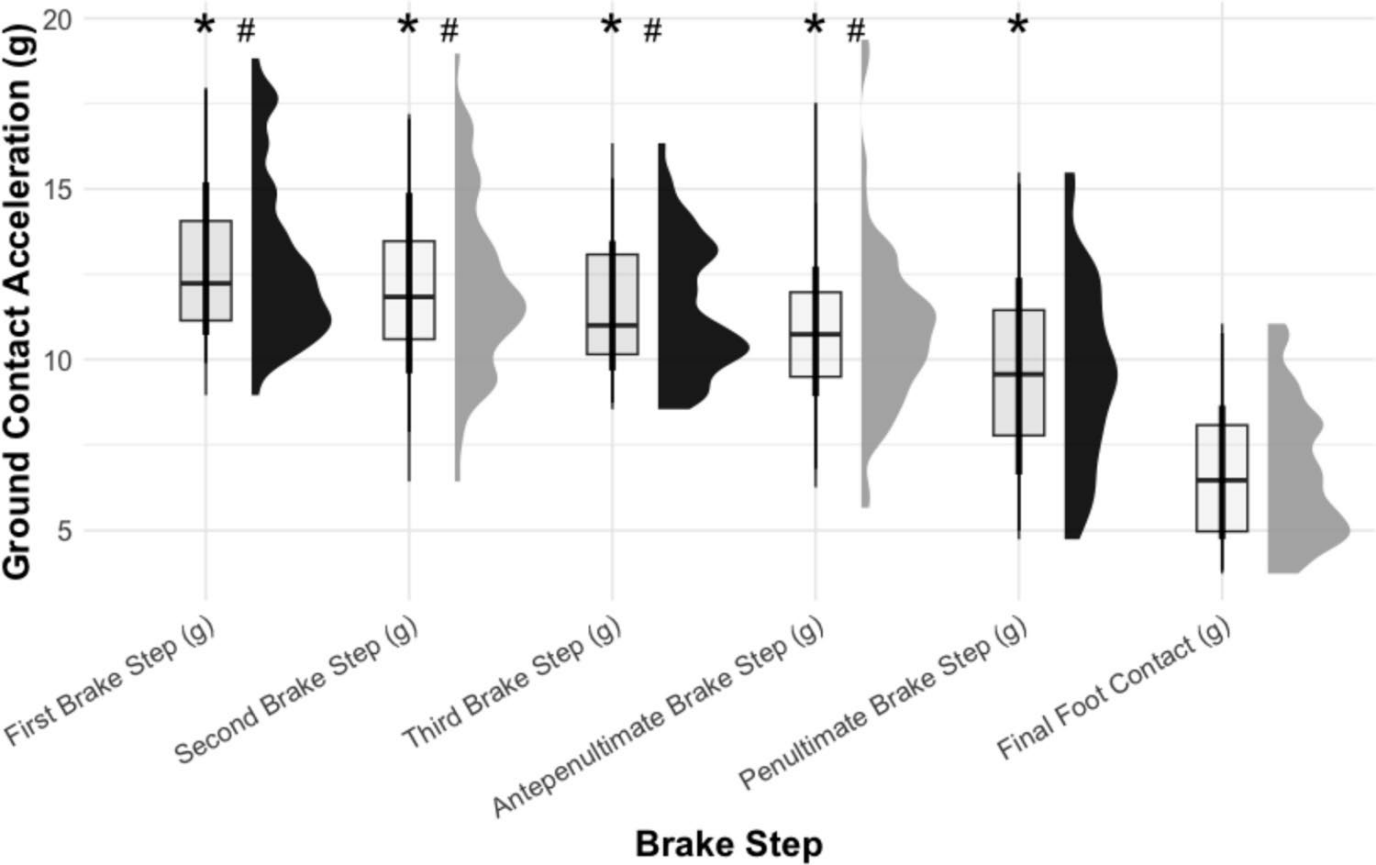
Comparison of braking step forces (i.e. acceleration; g) preceding a 180° turn in a 505 change of direction test captured using the Xsens inertial measurement unit system. Note: Athletes in this test can take around five braking steps prior to the final foot contact when turning. For the data used in the figure, the suit configuration “lower body with sternum” was selected in the Xsens MVN software (MVN Record 2023), with inertial measurement units placed around the anterior superior part of the foot, the tibia close to the knee, the middle of the lateral thigh, the posterior pelvis at the height of the anterior superior iliac spine, as well as the sternum. *Significant difference from final foot contact, ^#^significant difference from penultimate foot contact

One advantage of IMUs is their capability to provide insights on step-by-step forces encountered when decelerating, especially when large-scale force plates are not available. For example, Nedergaard et al. [90] used an accelerometer mounted on the upper torso to highlight the high forces encountered during the preparatory deceleration steps prior to turning and Gageler et al. [91] used accelerometers positioned on the ankle, knee, sacrum and upper torso to illustrate shock attenuation demands during decelerations with 6-m and 3-m stopping zones. Figure 3 illustrates comparisons of the braking step forces (g) preceding a 180° turn in a traditional 505 COD test captured using the Xsens IMU system. The data clearly highlight the heightened loading characteristics of the early deceleration steps, echoing the findings by Nedergaard et al. [90]. With regard to the magnitude of brake step peak forces (g), while reliable [50], further research is warranted to address the validity of the Xsens IMU system for quantifying peak brake step forces (g) during deceleration. Future research is also needed to investigate the reliability and validity of different IMU systems, sensor positionings (e.g. foot, shank, thigh, trunk mounted) and sampling frequencies for the purposes of simultaneously measuring whole body deceleration and step-by-step kinetic and kinematic data associated with each braking step.

### Advantages and Disadvantages of Different Technologies Used to Measure Deceleration

A summary of the advantages and disadvantages associated with each measurement technology for measuring deceleration is provided in Table 2. Practitioners and organisations can use Table 2 to help inform decisions around which technology device may be more suitable for their needs. Common considerations presented within Table 2 include portability, cost, sampling frequency, set-up time, post-processing time, indoor and/or outdoor usage, compatibility with different deceleration test types, efficiency for use with multiple athletes, type of data captured and ability to integrate with other technologies to capture additional data. For example, if a practitioner wants to just investigate the average deceleration obtained from a velocity time curve, technologies with lower sampling frequencies (i.e. 15–120 Hz) may suffice. However, if there is a need to obtain more details, such as those associated with step-by-step data (e.g. ground contact times, peak forces and loading rates), technologies with higher sampling frequencies (i.e. ≥ 120 Hz) would be required.

**Table 2:** Advantages and disadvantages of different technologies that can be used to assess deceleration

Technology	Advantages	Disadvantages
Laser	1. No pre-calibration required because of the distance being calculated from speed of light and time of flight from when laser emitted to being received (only zeroing) 2. Tracks specific target (i.e. lower back). Therefore, less sensitive to spurious reflections from other objects 3. Instantaneous feedback 4. Can be used indoors and outdoors to obtain the correct shoe-surface interaction 5. High sampling frequency (i.e. > 100 Hz)	1. Narrow targeting area requiring athletes to stay within a narrow linear running area. Therefore, restricted to deceleration measurement in linear tasks 2. Uses reflected pulsed infrared light to determine position of the athlete relative to the device. Therefore, to calculate deceleration requires double differentiation of distance 3. Cost
Radar	1. Wide measuring span 2. Quick to set up 3. Can be used indoors and outdoors to obtain the correct shoe-surface interaction 4. Sampling frequency of typical systems (i.e. 47–60 Hz) 5. 3D radar devices permit measurement of deceleration in multi-planar change of direction tests	1. Wide measuring span means care needs to be taken that moving objects are not within the range of the device 2. Some devices require long post-processing times, which can be time consuming 3. Multiple raters can affect reliability of measures when analysing data manually
Motorised resistance device	1. Can be used indoors and outdoors to obtain the correct shoe-surface interaction 2. Quick set-up (i.e. belt with cord attachment) 3. Easily integrated as part of a training session (i.e. invisible monitoring) that avoids the feeling of a “test session” 4. Instantaneous feedback 5. High sampling frequency (i.e. > 200 Hz)	1. Involves an external load, meaning deceleration is either assisted or resisted dependent on positioning of the motorised resistance device 2. External load in an assisted or resisted condition could change deceleration kinetics and kinematics from a natural unloaded condition 3. Cannot be used to measure an unloaded condition 4. Cost
Global navigation satellite system	1. Most teams have global navigation satellite system units and wear devices routinely in training, making testing of deceleration more efficient in training sessions 2. Can integrate invisible monitoring so deceleration assessment is integrated into training sessions (i.e. pitch-based warm-ups, conditioning and top-up sessions) 3. Multiple athletes can be assessed simultaneously 4. Possible for live feedback with some deceleration metrics 5. Global navigation satellite system units contain inertial sensors (i.e. accelerometer, gyroscope, magnetometer) that can be used to detect additional movement data	1. Requires individual trials to be extracted from session to conduct analysis 2. Accuracy can be affected by number of satellites detected 3. No immediate feedback for some deceleration metrics 4. Can only be used outdoors, meaning surface may be inconsistent 5. Lower sampling frequencies (i.e. < 25 Hz) in sport application makes accurate detection of rapid changes in velocity and identification of start/end of deceleration phase less accurate 6. Involves a wearable product, which can result in restricted athlete compliance 7. Cost when purchased for multiple users (i.e. team settings)
Video	1. Provides a visual record of performance for kinematic analysis relating to deceleration technique 2. Pose recognition and artificial intelligence-based software applications can provide analysis within a few minutes 3. Can be used indoors or outdoors to obtain the correct shoe-surface interaction 4. Cost-effective accessible feature on most smart phones and tablet devices with higher sampling rates possible (i.e. 120 Hz) 5. Allows a simultaneous assessment of the deceleration technique to identify possible technique deficits linked to sub-optimal performance and injury risk	1. Manual digitisation to attain centre of mass position and velocity can be time consuming with some systems 2. If a single camera is used, this needs to be positioned in a sagittal plane for assessment of whole-body deceleration metrics 3. When using automated pose recognition technology systems, there needs to be good contrast between the background and the athlete 4. Limited validity and reliability data for pose recognition and artificial intelligence-based software applications
Inertial measurement units	1. Not restricted to a laboratory with similar accuracy to laboratory-based motion cameras 2. Can be easily integrated with other technologies to provide more detailed insights into deceleration performance (e.g. step-to-step forces, ground contact times) 3. Can be used indoors and outdoors to obtain the correct shoe-surface interaction	1. Inertial measurement unit drift (i.e. gradual accumulation of errors in position, velocity or orientation) 2. Calibration procedures necessary prior to usage 3. Reliability in some cases decreases at higher movement speeds 4. Involves a wearable product, which can result in restricted athlete compliance 5. Cost

*3D* three-dimensional

## Key Variables Used to Assess Deceleration

### Whole Body Deceleration

**Fig. 4:**
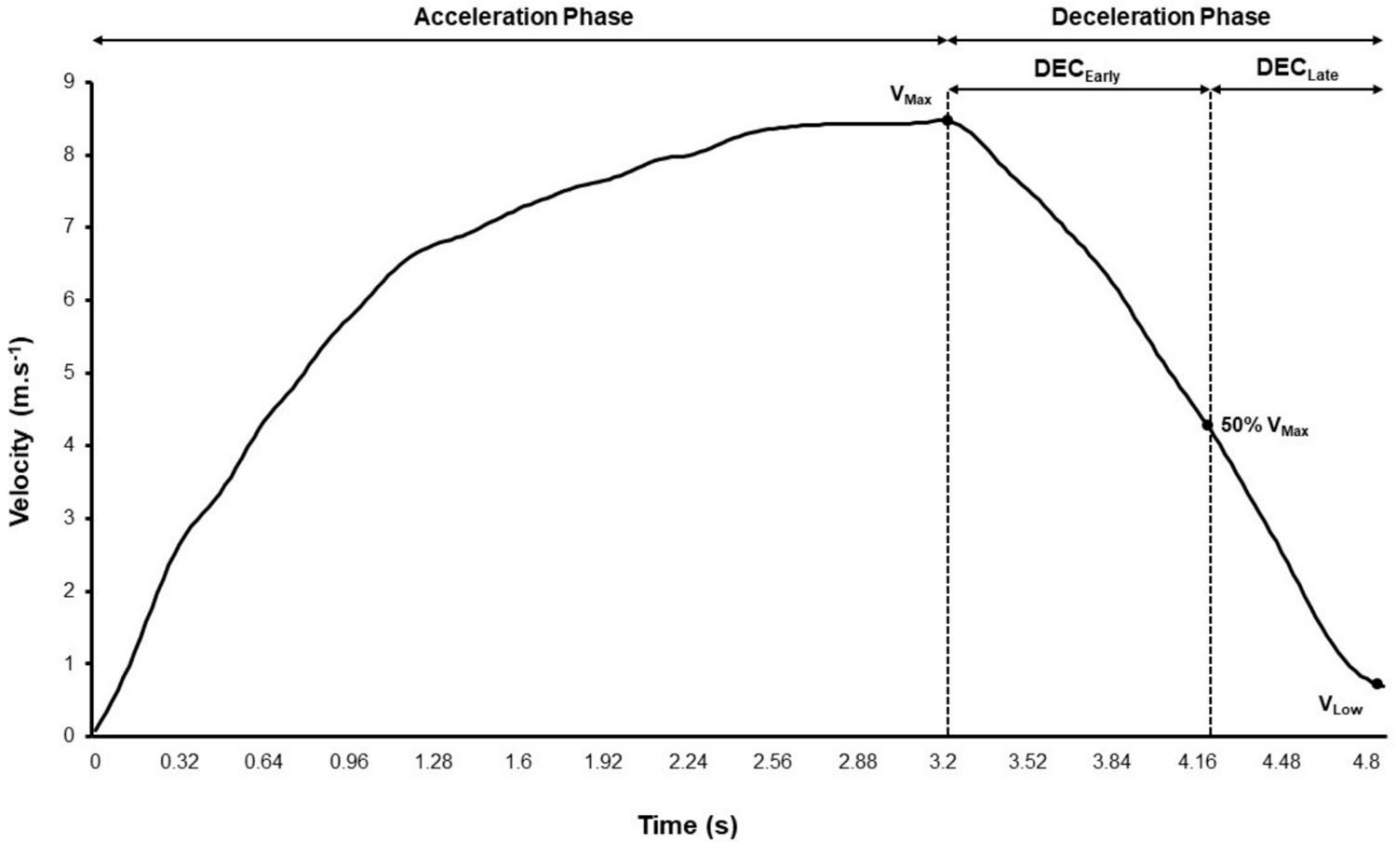
Velocity–time profile captured during an acceleration-deceleration ability (ADA) test. The start of the deceleration phase is defined using maximum velocity (*V*_Max_) with the end of the deceleration phase defined using the lowest velocity (*V*_Low_). Fifty percent of maximum velocity (50% *V*_max_) is used to identify the early (DEC_Early_) and late (DEC_Late_) deceleration sub-phases

For the purposes of assessing whole body deceleration performance, it is important to ensure accurate identification of the start and end of the deceleration phase. Based on current research and applied practices, we recommend using the timepoint immediately following maximum velocity to signify the start of the deceleration phase, and the lowest velocity attained upon stopping (i.e. zero velocity) or immediately prior to changing direction to signify the end of the deceleration phase (Fig. 4). The deceleration phase can be further sub-divided into the early and late deceleration sub-phases using the timepoint associated with 50% of maximum velocity. Analysing deceleration performance in these sub-phases is important because of the different biomechanical [18] and physical demands [46, 93] associated with decelerating (i.e. braking) from higher (i.e. early deceleration) compared to lower velocities (i.e. late deceleration). Additionally, athletes who have lower deceleration capabilities in the early deceleration sub-phase may be more prone to heightened deceleration requirements in the late deceleration sub-phase, which can be associated with inferior deceleration and COD performance and heightened lower limb forces and surrogates of injury risk, such as to the ACL, in the final foot step [8]. Further research is required to investigate the influence of different deceleration start-and-end phase criteria, such as those based upon a percentage or absolute threshold (e.g. percentage or unit decrease in velocity), foot interaction with the ground, and other metrics such as acceleration and its derivatives [50, 56]. For example, West et al. [56] compared use of a deceleration threshold method (i.e. deceleration starts from first value ≤ 1.5 m/s^2^) with set distance and peak velocity methods and reported the best inter-session reliability across key deceleration metrics (i.e. average deceleration, distance and time to stop) when using the deceleration threshold method averaged across the best of two trials.

Using the deceleration velocity–time profile attained from either a COD (Fig. 2) or ADA (Fig. 4) test, there are several metrics that can be used to evaluate an athlete’s whole-body deceleration performance. Table 3 provides a definition of these metrics, equations used to calculate them, and their importance for sports performance and injury-risk reduction.

**Table 3:** Definition of whole-body deceleration performance metrics, equations used to calculate them and their importance for sports performance and injury risk

Deceleration metric (unit)	Definition	Equation	Importance
Maximum velocity (m.s^−1^)	Peak velocity of the athlete recorded immediately prior to commencing deceleration	$V\mathrm{Max}= \frac{\Delta x}{\Delta t}$	Deceleration demands relative to the athlete’s approach velocity. Generally, deceleration demands are greater for faster athletes
Maximum momentum (kg/m.s^−1^)	Peak momentum of the athlete recorded immediately prior to commencing deceleration	$\mathrm{Max} P= (m x V\mathrm{Max})$	Deceleration demands relative to the athlete’s approach momentum. Generally, deceleration demands are greater for faster/heavier athletes, and thus should be taken into account when longitudinally monitoring and benchmarking
Average deceleration (m.s^−2^)	Average of all instantaneous deceleration values recorded across the deceleration phase	${\sum }_{V\mathrm{Min}}^{V\mathrm{Max}}\frac{(vf-vi)}{(tf-ti)}$	Ability to maintain high deceleration values across the entire deceleration phase
Maximum deceleration (m.s^−2^)	Maximum instantaneous deceleration value recorded across the deceleration phase	$\text{Max }\frac{(vf-vi)}{(tf-ti)}$	Highest maximum deceleration value generated by the athlete. Be aware, this could be linked to both better deceleration capacity and/or injury-risk (i.e. higher peak forces) and is only representative of one sample of data, which is highly influenced by sampling frequency and filtering approach
Average early deceleration (m.s^−2^)	Average of all instantaneous deceleration values across the early deceleration sub-phase (i.e. *V*_Max_ to 50% *V*_Min_)	${\sum }_{50\% V\mathrm{Max}}^{V\mathrm{Max}}\frac{(vf-vi)}{(tf-ti)}$	How quickly the athlete can decelerate within the initial steps of commencing deceleration. Indicates better ability to generate a high horizontally orientated braking impulse in early deceleration steps
Average late deceleration (m.s^−2^)	Average of all instantaneous deceleration values across the late deceleration sub-phase (i.e. 50% *V*_Max_ to *V*_Min_)	${\sum }_{V\mathrm{Min}}^{50\% V\mathrm{Max}}\frac{(vf-vi)}{(tf-ti)}$	Deceleration generated in the later steps of the entire deceleration phase. Whilst higher values would be important for performance, it is important to note that high deceleration values in this sub-phase could also be linked to injury risk (i.e. they are slamming on the brakes late, perhaps because of an inability to reduce speed within the early deceleration sub-phase)
Distance to stop (m)	Distance taken to stop during the deceleration phase	${\sum }_{V\mathrm{Min}}^{V\mathrm{Max}}\frac{1}{2} \left(vf-vi\right)*\left(tf-ti\right)$	Shorter distance to decelerate highly advantageous from a performance perspective (e.g. they can approach changes of direction at faster movement speeds because of less distance to stop). It is important to note this metric could be influenced by an athlete’s deceleration strategy and anthropometric characteristics (e.g. tall athlete with greater step lengths may require greater distance to stop when compared to an athlete with shorter leg lengths)
Time to stop (s)	Time taken to stop during the deceleration phase	${\sum }_{V\mathrm{Min}}^{V\mathrm{Max}}(tf-ti)$	Shorter time to decelerate would be highly advantageous from a performance perspective (e.g. they can approach changes of direction at faster movement speeds because of less time to stop)

*50% Vmax* 50% of maximum velocity, *f* final, *i* initial, *m* mass, *Max* maximum, *Min* minimum, *t* time, *v* velocity, *VMax* maximum velocity, *VMin* minimum velocity, *x* displacement

Tables 4 and 5 illustrate deceleration scores for whole body deceleration performance metrics measured across the entire deceleration phase in ADA and COD tests, respectively. The values provide practitioners with some normative data that can be used to compare deceleration performance outcomes amongst athletes for a variety of deceleration performance assessments and measurement devices. It is important to note, however, that the filtering of data differs between measurement devices and software applications, making comparisons between systems difficult. Therefore, for purposes of comparison, it is recommended to only compare values to the same measurement device and software application. Some of these metrics have also been used to calculate deceleration ratios to indicate athlete deceleration performance and braking strategies. For example, the average early deceleration (m/s^2^) can be divided by the average late deceleration (m/s^2^) to signify how quickly the athlete can decelerate within the initial braking steps of commencing deceleration [39]. A value closer to one would suggest a more balanced deceleration strategy or that the athlete can generate higher deceleration within the early horizontal deceleration sub-phase relative to the late horizontal deceleration sub-phase. For purposes of injury-risk reduction and evaluating rehabilitation and return-to-play programmes, a deceleration index has been proposed as an important measure of the rate at which a player can slow down relative to their ability to accelerate by calculating deceleration time relative to acceleration time [28], although it is feasible that other metrics (e.g. deceleration and acceleration; m/s^2^) could also be used. Whilst these ratios could help to highlight some potential strengths and deficiencies of the athlete, for the purposes of monitoring changes over time it is important to monitor the component parts, owing to there being more than one way the ratio could change and to minimise the measurement error that could be compounded by combining two component parts [94]. Further research is required to establish the reliability of these deceleration ratios and to establish their importance for performance, injury-risk reduction and rehabilitation purposes.

**Table 4:** Deceleration performance scores for whole body deceleration performance metrics measured across the whole deceleration phase in ADA tests. Values reported in mean ± standard deviation with co-efficient of variation percentage in bold and brackets

Study	*n*, sex	Sport (position)	Standard	Test	Device	*V*_Max_ (m/s)	DEC_Ave_ (m/s^2^)	DEC_Max_ (m/s^2^)	DEC_Early_ (m/s^2^)	DEC_Late_ (m/s^2^)	DTS (m)	TTS (s)
Chen et al. [43]	18 M	–	Collegiate	ADA5	Radar	5.33 ± 0.31 **(1.9)**	− 4.76 ± 0.44 **(1.7)**				3.02 ± 0.44 **(5.8)**	1.02 ± 0.11 **(3.3)**
Chen et al. [46]	57 (41 M, 16F)	Team sport	Youth	ADA5	Radar		− 3.97 ± 0.69				3.40 ± 0.62	1.18 ± 0.18
Chen et al. [43]	18 M	–	Collegiate	ADA10	Radar	6.59 ± 0.35 **(1.1)**	− 4.58 ± 0.37 **(1.6)**				4.97 ± 0.67 **(4.5)**	1.34 ± 0.14 **(3.7)**
Chen et al. [46]	57 (41 M, 16F)	Team sport	Youth	ADA 10	Radar		− 3.90 ± 0.77				5.41 ± 1.23	1.52 ± 0.30
Philipp et al. [39]	22 F	Softball	NCAA D1	ADA10^a^	Radar	6.20 ± 0.35 **(3.2)**	− 3.26 ± 0.30 **(7.6)**					1.46 ± 0.16 **(9.1)**
Philipp et al. [48]	11 F	Handball	Professional	ADA10	Radar	6.42 ± 0.34 **(3.0)**	− 3.65 ± 0.51 **(8.5)**	− 8.79 ± 1.17 **(8.1)**			6.03 ± 0.48 **(16.7)**	1.67 ± 0.16 **(10.4)**
Philipp et al. [49]	5 M	Basketball	Professional	ADA10^b^	Radar	6.53 ± 0.35	− 4.45 ± 0.77					1.33 ± 0.17
	5 M	Basketball	Professional	ADA10^c^	Radar	1.25 ± 0.39 **(2.9)**	− 3.29 ± 0.33 **(10.2)**					1.73 ± 0.19 **(10.0)**
Phillip et al. [50]	19 (14 M, 5F)	Team	Recreational	ADA10	IMU	4.43 ± 0.24	− 3.32 ± 0.41				4.17 ± 0.61	1.20 ± 0.18
Philipp et al. [52]	20 (15 M, 5F)	Team	Recreational	ADA10	IMU	4.45 ± 0.06 **(1.8**^**d**^**)**	− 3.29 ± 0.09 **(8.4**^**d**^**)**				4.14 ± 0.16 **(13.7**^**d**^**)**	1.20 ± 0.18 **(8.7**^**d**^**)**
				ADA10 (2% BM)	IMU	4.39 ± 0.06	− 3.23 ± 0.09				4.19 ± 0.15	1.23 ± 0.03
				ADA10 (4% BM)	IMU	4.38 ± 0.06	− 3.14 ± 0.09				3.90 ± 0.15	1.21 ± 0.03
Philipp et al. [47]	8 M	AF (CB)	NCAA D1	ADA10^a^	Radar	7.02 ± 0.26 **(3.1–3.6)**	− 5.06 ± 0.61 **(6.9–8.9)**	− 8.97 ± 0.99 **(7.7–8.0)**	− 4.74 ± 0.85 **(9.3–12.4)**	− 6.02 ± 1.09 **(11.3–12.2)**	5.10 ± 0.86 **(7.5)**	1.14 ± 0.15 **(6.0)**
	9 M	AF (ILB)	NCAA D1	ADA10^a^	Radar	6.66 ± 0.28	− 4.62 ± 0.45	− 8.75 ± 0.51	− 4.24 ± 0.56	− 5.28 ± 0.83	5.24 ± 0.51	1.26 ± 0.11
	8 M	AF (OLB)	NCAA D1	ADA10^a^	Radar	6.57 ± 0.17	− 4.76 ± 0.55	− 9.04 ± 1.08	− 4.51 ± 0.73	− 5.29 ± 0.61	4.81 ± 0.80	1.20 ± 0.14
	5 M	AF (QB)	NCAA D1	ADA10^a^	Radar	6.26 ± 0.27	− 4.52 ± 0.77	− 8.31 ± 0.36	− 4.25 ± 0.92	− 5.20 ± 0.55	4.60 ± 0.58	1.13 ± 0.08
	9 M	AF (RB)	NCAA D1	ADA10^a^	Radar	6.56 ± 0.38	− 4.72 ± 0.54	− 8.46 ± 0.79	− 4.38 ± 0.70	− 5.55 ± 0.60	4.86 ± 0.87	1.13 ± 0.16
	9 M	AF (SAF)	NCAA D1	ADA10^a^	Radar	6.73 ± 0.32	− 5.18 ± 0.74	− 8.85 ± 0.76	− 4.83 ± 0.92	− 5.87 ± 0.75	4.71 ± 0.82	1.11 ± 0.15
	24 M	AF (SP)	NCAA D1	ADA10^a^	Radar	6.35 ± 0.25	− 4.30 ± 0.59	− 7.66 ± 1.09	− 3.96 ± 0.66	− 4.98 ± 0.93	5.03 ± 0.91	1.27 ± 0.18
	5 M	AF (TE)	NCAA D1	ADA10^a^	Radar	6.31 ± 0.22	− 4.58 ± 0.65	− 8.27 ± 0.97	− 4.17 ± 0.65	− 5.34 ± 0.93	4.63 ± 0.70	1.17 ± 0.16
	11 M	AF (WR)	NCAA D1	ADA10^a^	Radar	6.90 ± 0.17	− 5.25 ± 0.64	− 8.57 ± 0.85	− 5.20 ± 0.70	− 5.43 ± 0.86	4.58 ± 0.55	1.08 ± 0.13
	5 F	LC (ATT)	NCAA D1	ADA10^a^	Radar	5.93 ± 0.20	− 4.08 ± 0.43	− 6.89 ± 1.64	− 3.54 ± 0.37	− 5.31 ± 0.81	4.84 ± 0.58	1.24 ± 0.17
	5 F	LC (DEF)	NCAA D1	ADA10^a^	Radar	5.89 ± 0.12	− 4.03 ± 0.49	− 7.29 ± 0.55	− 3.45 ± 0.49	− 5.30 ± 0.67	4.92 ± 0.63	1.27 ± 0.13
	1 F	LC (GK)	NCAA D1	ADA10^a^	Radar	5.97 ± 0.03	− 3.97 ± 0.08	− 6.59 ± 0.01	− 3.73 ± 0.14	− 4.32 ± 0.03	4.66 ± 0.11	1.32 ± 0.02
	9 F	LC (MF)	NCAA D1	ADA10^a^	Radar	6.03 ± 0.26	− 4.46 ± 0.55	− 7.43 ± 0.63	− 3.88 ± 0.63	− 5.73 ± 0.64	4.61 ± 0.66	1.16 ± 0.13
Li et al. [53]	42 M	Team	Trained/developmental	ADA 15	Radar	7.67 ± 0.29 **(9.8)**	− 4.21 ± 1.80 **(1.5)**	− 5.38 ± 2.41 **(1.6)**			6.99 ± 2.40 **(12.3)**	1.92 ± 0.36 **(6.4)**
Lin et al. [54]	32 M	Team	Trained/developmental	ADA 15	Radar	7.05 ± 0.86	− 4.87 ± 0.70	− 6.15 ± 0.52			6.87 ± 1.75	1.97 ± 0.35
Wei et al. [5]	32 (19 M, 13F)	Soccer	Collegiate	ADA 15	Radar	7.26 ± 0.46 **(2.1)**		− 6.48 ± 1.63 **(9.6)**				
Chen et al. [43]	18 M	-	Collegiate	ADA20	Radar	7.67 ± 0.29 **(1.5)**	− 4.12 ± 0.42 **(3.7)**				7.92 ± 0.91 **(6.4)**	1.72 ± 0.16 **(5.2)**
Harper et al. [29]	38 (29 M, 8F)	Team	University	ADA20^e^	Radar	7.17 ± 0.50 **(1.4**^**f**^**)**	− 4.39 ± 0.63 **(5.2**^**f**^**)**	− 8.40 ± 1.07 **(9.6**^**f**^**)**	− 3.77 ± 0.59 **(8.8**^**f**^**)**	− 5.53 ± 0.70 **(9.7**^**f**^**)**	6.53 ± 0.83 **(7.2**^**f**^**)**	1.47 ± 0.14 **(5.3**^**f**^**)**
Philipp et al. [39]	22 F	Softball	NCAA D1	ADA20^g^	Radar	7.06 ± 0.36	− 4.16 ± 0.36					1.70 ± 0.36
Jones et al. [44]	32 M	Soccer	Academy	ADA20	Radar	8.07 ± 0.26 **(1.1)**	− 4.33 ± 0.41 **(4.4)**	− 7.58 ± 0.89 **(6.0)**			7.86 ± 0.73 **(5.5)**	1.43 ± 0.14 **(5.5)**
					GPS	7.80 ± 0.30 **(1.4)**	− 4.29 ± 0.40 **(6.1)**	− 7.83 ± 1.01 **(5.5)**			8.25 ± 0.96 **(6.6)**	1.61 ± 0.15 **(5.8)**
Bustamante-Garrido et al. [23]	28 F	Field hockey	National	ADA30	Radar				− 3.37 ± 0.87			
West et al. [56]	20 (10 M, 10F)	Team	Regional	ADA30^h^	MRD	7.22 ± 0.64 **(1.2**^**i**^**)**	− 3.23 ± 0.79 **(7.0**^**i**^**)**				9.36 ± 1.98 **(11.7**^**i**^**)**	2.34 ± 0.46 **(7.8**^**i**^**)**
				ADA30^j^		7.19 ± 0.68 **(1.3**^**i**^**)**	− 3.91 ± 0.82 **(7.1**^**i**^**)**				5.98 ± 1.34 **(5.3**^**i**^**)**	1.83 ± 0.38 **(6.4**^**i**^**)**

*ADA* acceleration-deceleration ability, *ADA5* acceleration-deceleration ability test with 10 m/yard sprint approach, *ADA10* acceleration-deceleration ability test with 10 m/yard sprint approach, *ADA20* acceleration-deceleration ability test with 20 m/yard sprint approach, *ADA30* acceleration-deceleration ability test with 30 m/yard sprint approach, *AF* American Football, *ATT* attacker, *BM* body mass, *CB* corner back, *DEC*_*Ave*_ average deceleration, *DEC*_*Early*_ average early deceleration, *DEC*_*Late*_ average late deceleration, *DEC*_*Max*_ maximum deceleration, *DTS* distance to stop, *DEF* defender, *F* female, *GK* goalkeeper, *ILB* inside linebacker, *IMU* inertial measurement unit, *LC* lacrosse,* M* male, *MF* midfielder, *MRD* motorised resistance device, *NCAA D1* National Collegiate Athletics Association Division One, *OLB* outside linebacker, *QB* quarterback, *RB* running back, *SAF* safety, *SP* special teams player, *TE* tight end, *TTS* time to stop, *V*_*Max*_ maximum velocity, *WR* wide receiver

^a^Test distance 10 yards (9.14 m)

^b^Data from high-performing group

^c^Data from low-performing group

^d^Based on intra-session reliability data from unloaded condition

^e^Data reported from testing day 1

^f^Based on intra-session reliability data

^g^Test distance 20 yards (18.29 m)

^h^Data reported from peak velocity method on testing day 2

^i^Based on intra-session reliability data from the average of best two trials

^j^Data reported from deceleration threshold method on testing day 2

**Table 5:** Deceleration performance scores for whole body deceleration performance metrics measured across the whole deceleration phase in 180° change of direction tests. Values reported in mean ± standard deviation with co-efficient of variation percentage in bold and brackets

Study	*n*, sex	Sport (position)	Standard	Test	Device	*V*_Max_ (m/s)	DEC_Ave_ (m/s^2^)	DEC_Max_ (m/s^2^)	DEC_Early_ (m/s^2^)	DEC_Late_ (m/s^2^)	DTS (m)	TTS (s)
Eriksrud et al. [63]	16 M	Soccer	Elite	5-0-5 (AR3kg)	MRD	4.41 ± 0.23	− 5.38 ± 0.41	− 10.33 ± 0.92	− 3.74 ± 0.38	− 9.68 ± 0.81		0.82 ± 0.04
Petway et al. [65]	54 M	Basketball (Bigs)	NCAA Power 4	5-0-5^a^ (AR3kg)	MRD	4.25 ± 0.22		− 7.68 ± 0.75^b^				0.79 ± 0.11
	74 M	Basketball (Guards)	NCAA Power 4	5-0-5^a^ (AR3kg)	MRD	4.48 ± 0.29		− 8.13 ± 0.61^b^				0.75 ± 0.09
Westheim et al. [31]	21 (16 M, 5F)	Team	–	5-0-5 (AR3kg)	MRD	4.49 ± 0.17^c^ **(2.0**^**d**^**)**	− 5.97 ± 0.68^c^ **(5.4**^**d**^**)**	− 11.70 ± 1.30^c^ **(5.4**^**d**^**)**	− 4.08 ± 0.50^c^ **(6.1**^**d**^**)**	− 11.20 ± 1.32^c^ **(5.2**^**d**^**)**	2.14 ± 0.18^c^ **(5.8**^**d**^**)**	0.76 ± 0.07^c^ **(4.9**^**d**^**)**
Westheim et al. [31]	21 (16 M, 5F)	Team	–	10-0-5 (AR3kg)	MRD	5.98 ± 0.31^c^ **(1.4**^**d**^**)**	− 5.66 ± 0.58^c^ **(4.0**^**d**^**)**	− 12.00 ± 1.17^c^ **(5.4**^**d**^**)**	− 3.86 ± 0.49^c^ **(5.9**^**d**^**)**	− 10.80 ± 1.33^c^ **(7.4**^**d**^**)**	4.03 ± 0.41^c^ **(5.1**^**d**^**)**	1.06 ± 0.08^c^ **(3.4**^**d**^**)**
Eriksrud et al. [63]	16 M	Soccer	Elite	10-0-5 (AR3kg)	MRD	5.92 ± 0.23	− 5.25 ± 0.49	− 9.76 ± 0.95	− 3.80 ± 0.43	− 8.55 ± 0.75		1.13 ± 0.07
van den Tillar and Uthoff [62]	11 M	Handball	Regional	10-0-5	Laser	5.58 ± 0.31	− 5.32 ± 0.97					1.39 ± 0.07
	11 M	Handball	Regional	10-0-5 (AR3kg)	Laser	5.76 ± 0.23	− 4.89 ± 0.61					1.44 ± 0.08
	11 M	Handball	Regional	10-0-5 (AR6kg)	Laser	5.76 ± 0.44	− 4.46 ± 0.98					1.36 ± 0.28
	11 M	Handball	Regional	10-0-5 (AR9kg)	Laser	5.96 ± 0.31	− 4.55 ± 0.72					1.33 ± 0.17
	11 M	Handball	Regional	10-0-5 (RA3kg)	Laser	5.28 ± 0.29	− 5.49 ± 0.98					1.00 ± 0.13
	11 M	Handball	Regional	10-0-5 (RA6kg)	Laser	5.12 ± 0.27	− 5.91 ± 1.66					0.95 ± 0.23
	11 M	Handball	Regional	10-0-5 (RA9kg)	Laser	4.81 ± 0.29	− 5.40 ± 1.44					0.98 ± 0.31
Kaneko et al. [37]	24 M	Soccer	Youth	15-0-5	Laser			− 11.02 ± 1.72^e^				
	19 M	Soccer	Youth	15–0-5	Laser			− 9.60 ± 1.06^f^				
Philipp et al. [52]	20 (15 M, 5F)	Team	Recreational	15–0-5	IMU	4.49 ± 0.07 **(2.2)**	− 4.09 ± 0.07 **(5.5)**				5.33 ± 0.16 **(9.3)**	1.39 ± 0.05 **(5.5)**
				15-0-5 (2% BM)	IMU	4.45 ± 0.07	− 3.95 ± 0.07				5.25 ± 0.16	1.34 ± 0.05
				15-0-5 (4% BM)	IMU	4.42 ± 0.07	− 3.86 ± 0.07				5.03 ± 0.16	1.29 ± 0.05
Westheim et al. [31]	21 (16 M, 5F)	Team	–	15-0-5 (AR3kg)	MRD	6.82 ± 0.36^c^ **(1.4**^**d**^**)**	− 5.30 ± 0.65^c^ **(4.1**^**d**^**)**	− 11.80 ± 1.29^c^ **(5.1**^**d**^**)**	− 3.59 ± 0.49^c^ **(4.0**^**d**^**)**	− 10.30 ± 1.43^c^ **(8.3**^**d**^**)**	5.66 ± 0.57^c^ **(2.6**^**d**^**)**	1.29 ± 0.11^c^ **(3.3**^**d**^**)**
Eriksrud et al. [63]	16 M	Soccer	Elite	5-0-5 (AR3kg)	MRD	6.80 ± 0.26	− 4.98 ± 0.40	− 9.52 ± 0.66	− 3.67 ± 0.43	− 7.85 ± 0.56		1.36 ± 0.08
Philipp et al. [48]	11 F	Handball	Professional	15-0-5	Radar	6.54 ± 0.26 **(1.5)**	− 3.29 ± 0.58 **(11.8)**	− 9.53 ± 1.56 **(15.5)**			6.66 ± 1.41 **(11.40)**	1.45 ± 0.28 **(11.4)**
Phillip et al. [50]	19 (14 M, 5F)	Team	Recreational	15-0-5	IMU	4.46 ± 0.28	− 4.10 ± 0.29				5.38 ± 0.54	1.41 ± 0.18

*AR3kg* assisted-resisted load of 3 kg during test, *AR6kg* assisted-resisted load of 6 kg during test, *AR9kg* assisted-resisted load of 9 kg during test, *DEC*_*Ave*_ average deceleration, *DEC*_*Early*_ average early deceleration, *DEC*_*Late*_ average late deceleration, *DEC*_*Max*_ maximum deceleration, *DTS* distance to stop, *MRD* motorized resistance device, *RA3kg* resisted-assisted load of 3 kg during test, *RA6kg* resisted-assisted load of 6 kg during test, *RA9kg* resisted-assited load of 9 kg during test, *TTS* time to stop, *5-0-5* change of direction test with 5 m approach, *10–0-5* change of direction test with 10 m approach, *15-0-5* change of direction test with 15 m approach, *F* female, *M* male *IMU* inertial measurement unit

^a^Data reported for right foot turn

^b^Maximum deceleration calculated from the 0.5-s time interval with greatest average deceleration

^c^Data reported from session three to four

^d^Data reported from session four for right foot turn

^e^Data from fast group (based on time from 0 to 10 m of 15-0-5 COD test)

^f^Data from slow group (based on time from 0 to 10 m of 15-0-5 COD test)

### Deceleration Technique

Because of very large forces that are generated and required to be effectively attenuated in intense decelerations, the technical ability to decelerate can have significant implications for performance, rehabilitation and injury-risk reduction programmes. It is therefore important to identify and measure kinematic variables of deceleration technique that best associate with superior performance and injury-risk reduction. For example, athletes with lower deceleration movement quality scores (i.e. knee flexion: “shock absorption”, frontal plane knee projection angle: “limb stability”, pelvis angle: “pelvis stability”, lateral trunk angle: “trunk stability” and hip and knee flexion: “movement strategy”) have been reported to have higher knee joint loading (i.e. knee abduction moment) during the final foot contact of a 10-m maximal sprint-to-stop ADA task [89]. Using the antepenultimate (i.e. two steps prior to stop/COD), penultimate (i.e. one step prior to stop) and ultimate foot contact (i.e. final footstep) of a sprint-to-stop ADA task, key kinematic variables of a deceleration technique in the sagittal and frontal plane are illustrated in Tables 6 and 7, respectively.

**Table 6:** Key kinematic variables of deceleration technique captured in the sagittal plane using a two-dimensional video


	Variable	Importance
1	Touch down distance (m) or leg placement angle (°)	Foot is placed in front of COM to increase horizontal braking impulse (force × time). Could be normalised to stature to permit a fairer comparison between athletes of different stature. Notice in earlier braking steps, the point of contact is with the heel to maximise braking effect. The leg placement angle is the angle of the line connecting the COM to the ankle relative to the downward vertical; the larger the angle in front of the COM the higher the potential for greater braking impulse
2	Peak hip and knee flexion (°) and joint angular velocity (s)	Hip and knee flexion (i.e. co-flexion) during each foot contact helps to ensure the hips and trunk are positioned behind the lead foot braking leg during ground contact, enabling braking forces to be applied for longer, leading to greater reduction in momentum. Hip and knee flexion permits mechanical energy absorption and shock attenuation across lower body musculoskeletal structures helping to reduce potential tissue damage. Joint angular velocity is an indicator of loading severity and should be controlled to prevent excessive knee flexion. Greater knee flexion velocities are indicative of less muscular control (relative to the horizontal velocity at foot contact)
3	Step number (*n*), length (m) and frequency (Hz)	Step kinematics alter through shorter step lengths and greater step frequency. Notice at touchdown of the penultimate and ultimate foot contact, the rear foot is still in contact with the ground providing a dual foot stance that leads to greater stability, load sharing, longer braking times (no propulsion phase) and thus greater braking impulse. The number of braking steps will increase with greater approach velocities in order to reduce higher forward momentum. Those with reduced braking capacity will require a greater number of braking steps to reduce momentum because of lower braking and muscle force-generating capacities
4	Shin angle (°)	Shin angle is reflective of orientation of force application. To decelerate, a negative shin angle is required to ensure anterior force application (i.e. horizontal braking impulse). A less negative shin angle is more reflective of a cautious upright braking strategy requiring more braking steps to reduce momentum
5	Trunk angle (°)	Ideally, the trunk should ‘lean back’ or at the very least be upright at touchdown in each foot contact to help shift the COM further behind the foot to increase the braking impulse. Notice this is more pronounced in the antepenultimate foot contact, and thus, important for early deceleration. Excessive forward trunk flexion combined with an extended knee joint places the hamstrings in a lengthened state, which is why adopting a reclined or upright trunk and a partially flexed knee are recommended. Forward trunk flexion should be regarded as a compensatory strategy rather than a desired movement that comes into play when insufficient braking has occurred prior to final foot contact
6	Knee flexion on touchdown (°)	A partially flexed knee (approximately 30°) in the sagittal plane at touchdown helps lower multi-planar knee joint loads and reduce hamstring stretch loads. The athlete should have the capacity to resist excessive knee flexion to avoid prolonged ground contact times, but sufficient flexion to lower the COM and create stability in dual support
7	COM height (m)	Flexion of the ankle, knee and hip (i.e. triple flexion) and the absence of a flight phase leads to a lowering of the COM. Coupled with periods of dual foot support illustrated in the penultimate and ultimate foot contacts allows a more stable position (i.e. increased base of support). Lowering the COM helps to prevent forward rotation about the lead foot braking limb, permitting greater and prolonged anterior foot placements ahead of COM. Could be normalised to stature to permit fairer comparison between athletes of different stature

*COM* centre of mass

**Table 7:** Key kinematic variables of the deceleration technique captured in the frontal plane using a two-dimensional video


	Variable	Importance
1	Knee abduction at touchdown and at peak knee flexion (°)	Initial and peak knee abduction angles could lead to elevated knee joint loading and stress to connective tissues. Increased knee abduction angle could also be reflective of reduced hip and trunk control/strength, or as a result of compensatory movements made at the hip and trunk to counteract insufficient strength of the quadriceps to generate the internal knee extension moment necessary to counteract the external knee flexion moment arising from the braking ground reaction force
2	Frontal plane pelvic alignment (°)	Neutral pelvic alignment enables optimal trunk alignment, reducing the chance of elevating multiplanar knee joint loads
3	Frontal plane trunk alignment (°)	An upright trunk in the frontal plane allows better alignment of the ground reaction force through the lower limb and may help to prevent elevated multiplanar knee joint loads. Excessive forward trunk rotation around the hip could increase the demand on hamstrings, leading to increased potential of hamstring strains

## Implications for Practice and Future Research

### Performance Enhancement

The future evolution of match play in MDS sports will likely demand players to perform more frequent high-intensity decelerations requiring an ability to generate and tolerate high braking forces repeatedly [27]. Accurately assessing deceleration, therefore, is a crucial requirement to ensuring athletes have the capability to perform efficient and rapid changes in speed and direction necessary to close down and create spaces frequently during match play. By evaluating whole body deceleration performance alongside the athlete’s deceleration technique, practitioners can identify areas for improvement and prescribe individualised training programmes to enhance deceleration performance and mitigate potential injury risk. For example, this could be targeting specific strength (e.g. eccentric, reactive) or technical qualities (e.g. lowering of COM, optimising knee flexion, trunk control and foot positioning) underpinning deceleration performance [95]. Future research is needed to examine the effectiveness of different training interventions on enhancing measures of deceleration performance. In designing these interventions, practitioners could consider the recommendations and guidelines presented in the Braking Performance Framework that summarises training methods that could target the currently known determinants of deceleration ability [95].

### Rehabilitation and Injury-Risk Reduction

Deceleration assessment has been identified as a missing link in injury rehabilitation [28] and an important process in evaluating an athlete’s readiness to return to sport following injury [28, 96]. The assessment, monitoring and subsequent training of deceleration have also been highlighted to be a potential ‘vaccine’ for sports-related injuries [9]. For example, between 32 and 66% of non-contact ACL injuries in soccer have been reported to occur during a defensive pressing scenario, when whole body deceleration from high velocity precedes a directional change [19–22]. Therefore, assessing and training deceleration ability could be an important modifiable risk factor for reducing ACL injury risk [21] and other soft-tissue injuries, such as calf [97], hamstring [98] and rectus femoris [99] strains that have also been associated with deceleration manoeuvres. As female athletes have been reported to be 3.5 times more likely to sustain an ACL injury than male athletes [100], deceleration assessment and training may be particularly important for this population [101]. Future research is needed to obtain normative deceleration profiles in various populations and to investigate the importance of deceleration for rehabilitation and injury-risk reduction purposes.

## Conclusions

This article provides practitioners with methodological and practical considerations for assessing deceleration in an applied field-based environment. We highlight a range of different protocols (i.e. COD and ADA tests) and measurement technologies (i.e. radar, laser, video, GNSS, IMUs and MRDs) that can be used by practitioners to evaluate deceleration and some of the advantages and disadvantages of each. Key metrics associated with whole body deceleration performance and the kinematics underpinning the deceleration technique (i.e. movement quality) are highlighted. Given the performance, health and injury-risk implications associated with deceleration, assessment of this movement skill should be given high priority within any athlete multi-disciplinary support system.
